# Case Report: Diagnosing and treating gallbladder neuroendocrine neoplasms through comparative analysis: a case series and literature review

**DOI:** 10.3389/fonc.2025.1606850

**Published:** 2025-06-16

**Authors:** Jiayao Zhang, Jiandong Li, Yuxing Dong, Baochun Lu

**Affiliations:** ^1^ Department of Hepatobiliary and Pancreatic Surgery, Shaoxing People’s Hospital, Shaoxing, China; ^2^ School of Medicine, Graduate School, Zhejiang University, Hangzhou, China

**Keywords:** neuroendocrine neoplasms, neuroendocrine carcinoma, gallbladder, surgery, adjuvant therapy

## Abstract

**Background:**

Gallbladder neuroendocrine neoplasms (GB-NENs) are exceedingly rare in clinical practice. To date, no large-scale, multicenter prospective studies have been conducted on this disease, resulting in a lack of established diagnostic and therapeutic experience or consensus. This case series reports seven GB-NEN patients who underwent different treatment modalities with varying outcomes. By integrating our institutional experience with previous literature, we aim to provide some therapeutic recommendations for GB-NEN patients.

**Methods:**

The clinicopathological data of seven GB-NEN patients treated at our institution between June 2013 and June 2024 were retrospectively analyzed, with a focus on their treatment courses.

**Results:**

Seven GB-NEN patients did not exhibite specific clinical manifestations or distinctive imaging features. All patients underwent surgical intervention, including radical resection in four cases. The overall survival ranged from 3 to 55 months, with a median survival of 19 months.

**Conclusion:**

GB-NENs are highly aggressive and associated with poor prognosis. We recommend: 1) Radical cholecystectomy as the primary treatment for resectable GB-NENs; 2) Platinum-based chemotherapy as the first-line regimen, with close monitoring for drug resistance; 3) Early assessment of chemosensitivity to guide further treatment decisions, postoperative chemotherapy combined with adjuvant therapies may improve surgical efficacy.

## Introduction

Primary gallbladder neuroendocrine neoplasms (GB-NENs) represent a rare subset of neuroendocrine tumors (NETs), exhibiting a predilection for female individuals, comprising approximately 0.5% of all NETs, while gallbladder neuroendocrine carcinomas (GB-NECs) constitute 2.1% of all gallbladder malignancies based on data from the U.S. Surveillance, Epidemiology, and End Results (SEER) database (1, 2). GB-NENs are rarely encountered in clinical practice since the gallbladder is devoid of enterochromaffin cells from which the neuroendocrine neoplasia originates. Furthermore, these neoplasms often present without symptoms initially and have no specific tumor markers, resulting in frequent preoperative misdiagnosis. Histopathological analysis remains the diagnostic gold standard. No standardized clinical guidelines or consensus exist for GB-NENs; thus, management typically mirrors protocols for gallbladder cancer or other NENs. Surgical resection remains the cornerstone of treatment, with platinum-based chemotherapy regimens constituting the first-line systemic treatment. Adjuvant therapy is considered a potential survival-prolonging strategy for advanced-stage GB-NENs, which often present with distant metastases (**Supplementary Figure 1**) (3).

## Case series presentation

**Figure 1:**
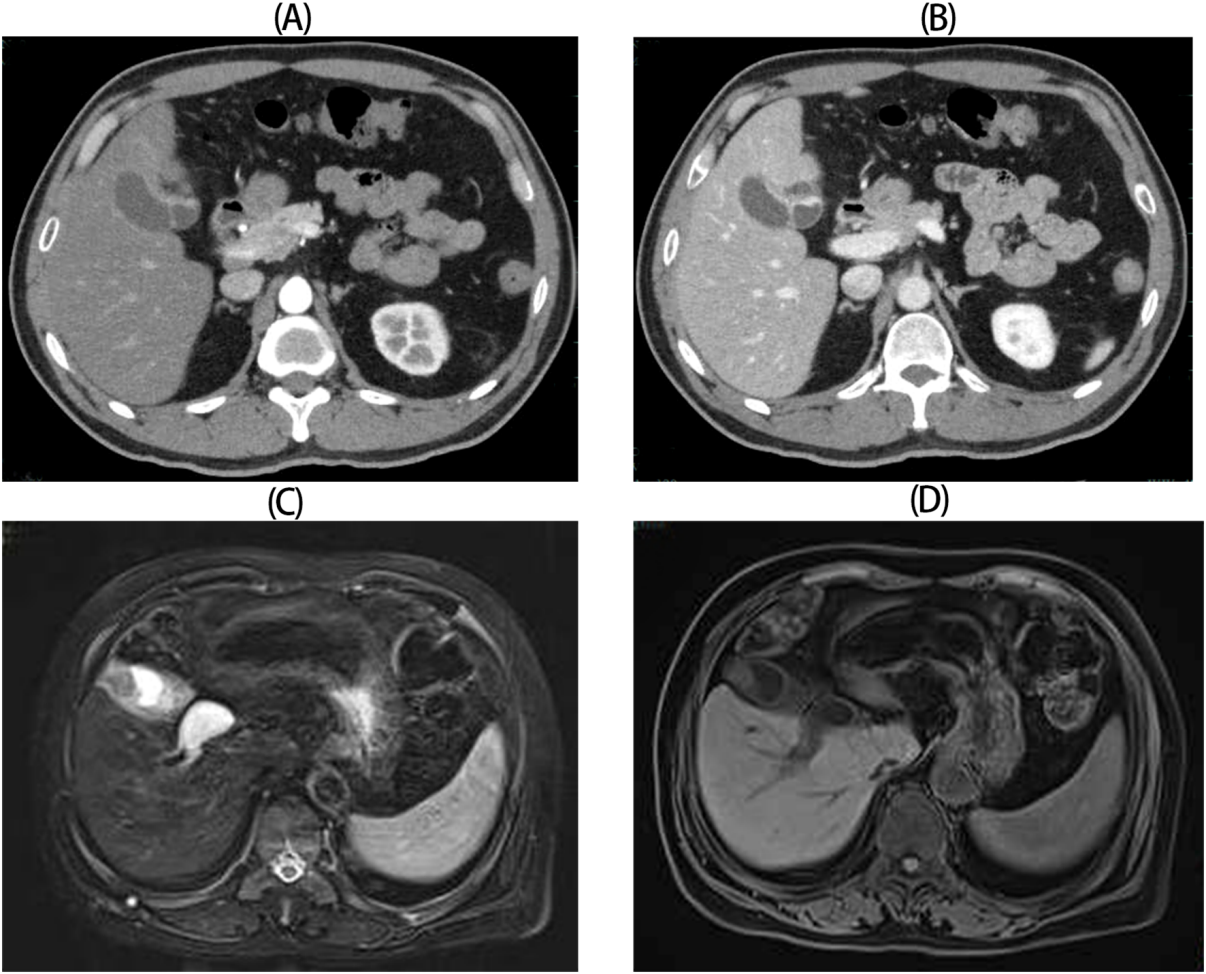
Radiologic images for GB-NENs. **(A, B)** Case 6: Irregular gallbladder wall thickening with arterial and venous phase enhancement (CT). **(C, D)** Case 5: An irregular, patchy soft-tissue signal at the gallbladder fundus (MRCP).

This study retrospectively analyzed clinical characteristics and treatment outcomes of seven GB-NEN cases treated at our institution, comprising three male and four female patients, with a median age of 64 years (range: 52–77). None exhibited disease-specific clinical manifestations at presentation. Four patients reported epigastric pain, two cases were detected during routine examinations, and one presented with jaundice due to bile duct involvement. Six patients had no evidence of associated hereditary syndromes (e.g., multiple endocrine neoplasia type 1) but a history of cholelithiasis complicated by cholecystitis. Except for one patient with elevated serum CA19–9 levels, all other biomarkers were within normal ranges(**Table 1**). Preoperative imaging uniformly revealed diffuse or focal gallbladder wall thickening. Among these, four cases were initially misdiagnosed as benign conditions (e.g., acute/chronic cholelithiasis or adenomyomatosis), whereas the remaining three exhibited space-occupying lesions, suggesting malignancy (**Figure 1**).

**Table 1:** Preoperative characteristics of 7 GB-NEN patients.

Case	Sex	Age	Clinical presentation	Gallstones	CA19-9	CEA	CA125
		(year)			(U/ml)	(U/ml)	(U/ml)
1	M	71	Right upper quadrant pain	Yes	N	N	N
2	M	64	Right upper quadrant pain	Yes	N	N	N
3	F	54	Incidental finding	Yes	N	N	N
4	F	64	Epigastric pain + jaundice	Yes	102.04	N	N
5	F	68	Incidental finding	Yes	N	N	N
6	M	52	Right upper quadrant pain	No	N	N	N
7	F	77	Right upper quadrant pain	Yes	N	N	N

M, male; F, female; N, normal.

Seven patients underwent surgical intervention. Three patients received radical cholecystectomy for gallbladder cancer based on preoperative diagnosis: Case 6 transitioned from etoposide-cisplatin (EP) to irinotecan-cisplatin (IP) due to treatment intolerance, supplemented with anlotinib. After six chemotherapy cycles developed bone marrow suppression, treatment was changed to penpulimab combined with anlotinib. The patient ultimately died of tumor recurrence 40 months postoperatively. Case 7 received only one EP cycle before refusing further chemotherapy. Liver metastases were detected at 2 months postoperation, with death occurring at 10 months. Case 5 exhibited unexpected 55-month disease-free survival (DFS) without adjuvant treatment. One patient received pancreaticoduodenectomy for distal bile duct involvement: Case 4 completed six gemcitabine-oxaliplatin (GMOX) cycles without subsequent adjuvant therapy and liver metastases emerged at 12 months, subsequent treatments(transarterial chemoembolization, EP chemotherapy, and lenvatinib plus camrelizumab) all proved ineffective, with death at 23 months postoperation. Two patients underwent simple cholecystectomy: Case 3 developed hepatic and distant metastases at 7 months, dying at 15 months. Case 2 was lost to follow-up. Case 1 with preexisting hepatic metastases underwent palliative resection without additional treatment (3-month survival) (**Table 2**).

**Table 2:** Surgical and Clinicopathological characteristics of 7 GB-NEN patients.

Surgical and prognostic					
Case	Surgical approach	Margin	TNM stage	Adjuvant Therapy	Survival (months)
1	Palliative cholecystectomy	R1	T4N1M1	None	3
2	Simple cholecystectomy	R0	T1N0M0	None	/
3	Simple cholecystectomy	R0	T2N0M0	None	15
4	Pancreaticoduodenectomy	R0	T3N0M0	Chemo + Immuno + Targeted	23
5	Radical cholecystectomy	R0	T2N0M0	None	55
6	Radical cholecystectomy	R0	T2N1M0	Chemo + Immuno + Targeted	40
7	Radical cholecystectomy	R0	T4N0M0	Chemotherapy	12

Clinicopathological characteristics								
Case	Tumor Location	Tumor Size cm	CgA	Syn	CD56	Ki-67(%)	Grade	Histological Type
1	Body	3.0*2.0*2.0	+	+	+	80%	G3	SCNECs
2	Body	0.9*0.5*0.2	+	+	/	1%	G1	/
3	Body	1.0*1.0*0.5	+	+	+	20%	G3	LCNECs
4	Neck	2.0*2.0*0.3	+	+	+	50%	G3	LCNECs
5	Fundus	2.0*1.0*0.5	+	+	+-	50%	G3	LCNECs
6	Neck	2.5*1.3*0.8	+	+	+	70%	G3	LCNECs
7	Body	2.0*2.0*1.0	+	+	+	60%	G3	LCNECs

**Figure 2:**
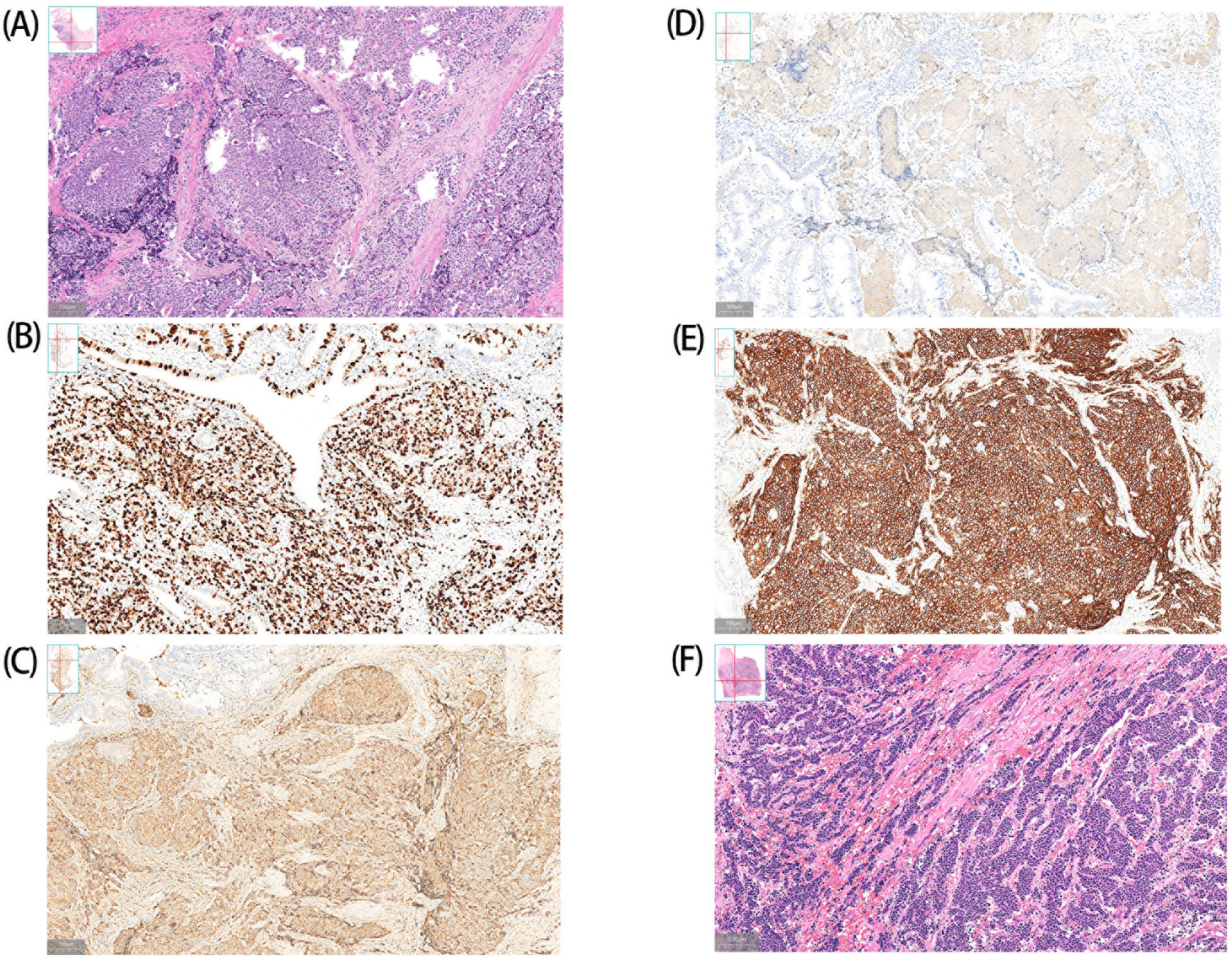
Histopathologic findings of GB-NENs. A (Case 6) and F (Case 1) show LCNECs and SCNECs, respectively. Case 6: Immunohistochemical analysis demonstrates positive staining for CgA **(C)**, Syn **(D)** and CD56 **(E)**, with a Ki-67 proliferation index of approximately 70% **(B)**.

Postoperative histopathological examination confirmed the diagnosis of GB-NENs in all cases, with predominant tumor localization in the gallbladder body(diameter range: 0.5–3 cm; invasion depth: 0.2–2 cm). Immunohistochemical profiling confirmed universal Ki-67 positivity, with proliferation indices >20% in six cases. According to the World Health Organization (WHO) 2019 classification, NETs are graded based on Ki-67 proliferation index as follows: G1 (<3%), G2 (3-20%), and G3 NEC (>20%) (4), so most patients were already diagnosed with NEC at presentation. The majority of cases represented large-cell neuroendocrine carcinoma(LCNECs), with only one exception being small-cell neuroendocrine carcinoma(SCNECs). By definition, a neoplasm can be qualified as mixed neuroendocrine-non-neuroendocrine neoplasms(MiNEN) when a neuroendocrine or a non-neuroendocrine component is morphologically and immunohistochemically recognizable and constitutes ≥ 30% of the tumor burden (5). Pathological analysis revealed adenocarcinoma components (7-10% of tumor) in five cases, which did not meet the diagnostic threshold for MiNEN. Uniform immunoreactivity was observed for three established neuroendocrine markers: chromogranin A (CgA), synaptophysin (Syn), and CD56, aligning with existing literature (4) (**Table 2**, **Figure 2**).

## Discussion

The preoperative diagnosis of GB-NENs remains a significant clinical challenge, with definitive confirmation typically achieved only through postoperative pathological examination of cholecystectomy specimens. Due to sampling limitations, percutaneous biopsy is not recommended. An overview of the 2022 WHO Classification of Neuroendocrine Neoplasms stated that CgA, Syn, and insulinoma-associated protein 1 (INSM1) represent general neuroendocrine markers and recommended the simultaneous use of Syn and INSM1, as this combination can reliably identify the neuroendocrine component (both NET and NEC) in nearly all cases. Notably, CgA staining may be negative or only focally positive in NEC components (6). Case 6 immunohistochemistry results were consistent with the above findings, showing strong Syn positivity but weaker CgA staining. Radiological findings provide supportive but non-definitive diagnostic value. A well-defined margin and intact overlying mucosa help differentiate GB-NENs from carcinomas (7). This distinguishing pattern is similarly observed when comparing gastric NENs to adenocarcinomas (8). Suspicious radiological characteristics warranting consideration of GB-NENs include GB-replacing masses, extensive growth patterns or bulky lymph node metastases at initial presentation (7, 9). In differential diagnosis, dual-energy CT plays a pivotal role in distinguishing between benign and malignant gallbladder pathologies (10). Quantitative parameters (iodine concentration and spectral curve characteristics) demonstrate significant differences between NENs and adenocarcinomas, thereby enhancing preoperative diagnostic accuracy. For non-emergency cases, gallium-68 or FDG-labeled PET imaging offers superior diagnostic precision. While histopathology remains the diagnostic gold standard, further investigation of GB-NEN-specific pathological signatures is warranted. Novel histopathological correlates, such as well-defined margins (neuroendocrine cells reside in subepithelial lamina propria which help to preserve overlying mucosa) (7), may enhance imaging interpretation and preoperative diagnostic accuracy. This integrated diagnostic approach, combining advanced pathological characterization with refined imaging criteria, represents a promising direction for improving GB-NEN diagnosis prior to surgical intervention.

NENs are thought to arise from neuroendocrine cells distributed systemically, but since the normal gallbladder lacks enterochromaffin cells from which the neuroendocrine neoplasia originates, the cellular origin of GB-NENs remains controversial, with two predominant hypotheses currently proposed. The first suggests GB-NENs may originate from gastric/intestinal metaplasia of gallbladder mucosal epithelium induced by chronic inflammation (11, 12), while the second posits that GB-NENs could derive from transdifferentiation of gallbladder adenocarcinoma (4). Emerging research increasingly favors the transdifferentiation hypothesis: 1) Existing literature demonstrates that the majority of reported GB-NEN cases are associated with gallstone-related chronic inflammation (4), a finding that aligns with our observed incidence of 85.71%, which is also a well-established risk factor for gallbladder cancer (12, 13). Chirag et al. have further delineated the inflammatory mechanisms driving gallbladder carcinogenesis (14). Both GB-NETs and carcinomas exhibit gastric/intestinal metaplasia and CgA-positive cells (11), suggesting a potential common precursor lesion. 2) Persistent histological alterations frequently drive the acquisition of molecular changes. A recent study unveiled the mutation landscape of 15 cases of GB-NENs by using whole-exome sequencing (WES) technology and found that TP53 showed the highest mutation frequency (73%, 11/15) (15). Intriguingly, TP53 mutations are also the earliest and most prevalent genomic event in gallbladder cancer (14, 16), implicating overlapping oncogenic pathways. 3) Emerging insights into GB-MiNENs, coupled with molecular evidence demonstrating intimate associations between neuroendocrine and non-neuroendocrine components in intestinal counterparts and strongly supporting a monoclonal origin asthe most frequent genetic event (17), which lend credence to the hypothesis of shared cellular origins and subsequent lineage divergence in GB-NENs (18). Elucidating GB-NET origins could inform risk mitigation strategies and refine mechanistic understanding of tumor evolution to guide targeted therapies, though further investigation is needed to fully resolve the cellular and molecular mechanisms underlying these processes.

The low incidence of GB-NENs has limited current literature predominantly to case reports and small case series, no large-scale, multicenter prospective studies have been conducted. Consequently, prognostic predictors remain poorly characterized, and no internationally standardized management strategies exist. Given that all NENs originate from neuroendocrine cells and emerging evidence suggesting GB-NENs may share a common origin with gallbladder cancer, we propose that treatment strategies for GB-NECs may be partially informed by protocols for gallbladder cancer and NENs at other sites. Furthermore, the widespread adoption of WES has enabled the identification of novel driver mutations and pathways potentially involved in GB-NENs pathogenesis, revealing promising therapeutic targets and informing the application of existing targeted therapies:

## 1 Radical cholecystectomy recommended for surgically eligible GB-NENs regardless of grade or stage

There is no consensus on the optimal treatment for GB-NENs (4). Surgical resection remains the primary therapeutic approach, with options including simple cholecystectomy, radical cholecystectomy, and palliative cholecystectomy. Radical cholecystectomy is the standard surgical procedure for gallbladder cancer and is also the mainstay of treatment for GB-NENs. Shekhar et al. conducted a retrospective analysis of data from the SEER database (1973–2016) encompassing all GB-NEN patients and found that those who underwent surgery had significantly better survival outcomes (mean survival: 111.0 ± 8.3 vs. 8.3 ± 1.2 months, P < 0.01) (19). In our case series, four patients underwent radical surgery and achieved relatively favorable survival. A systematic review of surgical management for T1-stage gallbladder cancer indicated that simple cholecystectomy is sufficient for T1a lesions, with no evidence supporting superior outcomes for T1b patients receiving radical cholecystectomy (20). Nevertheless, we recommend radical resection with regional lymphadenectomy when clinically feasible, as GB-NENs demonstrate markedly higher aggressiveness and poorer prognosis compared to conventional gallbladder cancers. A retrospective study have revealed that lymph node metastasis (N2) occurs significantly more frequently in GB-NEC than in gallbladder cancer (70.0% vs. 34.0%; P < 0.05) (2). Furthermore, EMS of GB-NEC identified ZFHX3 as the second most frequently mutated gene (15). Given that ZFHX3 mutations in endometrial tumors are associated with higher tumor grade and increased lymphovascular space invasion (21), it raises the question of whether such mutations also contribute to elevated lymphatic metastasis risk in GB-NENs.

## 2 Platinum-based first-line chemotherapy optional, with adjuvant therapies for potential prognostic improvement

### 2.1 Role of chemotherapy

Current evidence regarding adjuvant chemotherapy for GB-NENs remains inconclusive. Some studies report improved median overall survival (OS) and DFS with postoperative chemotherapy (11, 22). For instance, Case 6 in our series, initially presenting with N1 lymph node metastasis, achieved 40 months of survival following multimodal therapy, including EP, IP with anlotinib, and pembrolizumab-anlotinib regimens, surpassing the reported median survival of 23.2 months (19). However, a decade-long multicenter study by Wang et al. found no statistically significant prognostic benefit from adjuvant chemotherapy (4). Notably, Case 5 in our cohort remained recurrence-free for 55 months after radical cholecystectomy without chemotherapy. We posit that chemotherapeutic responsiveness typically manifests early; discontinuation should be considered for non-responders to avoid severe adverse events, such as hepatorenal toxicity or leukopenia (3, 23).

### 2 Chemotherapy regimens

The rarity of GB-NENs precludes large-scale prospective or retrospective studies, leaving no standardized chemotherapy protocol. Platinum-etoposide combinations, extrapolated from small-cell lung cancer treatment paradigms and supported by small retrospective studies demonstrating chemosensitivity in NENs (objective response rate: 40-70%) (23–25), have become the consensus first-line regimen. Updated European Neuroendocrine Tumor Society Guidelines(ENETS) guidelines recommend carboplatin or irinotecan as potential substitutes for cisplatin and etoposide, respectively (26). A retrospective analysis by Hiroo et al. indicate comparable progression-free survival (PFS) (95% CI: 3.1–7.0 vs. 3.5–6.3, P = 0.781) and OS (95% CI: 11.2–14.6 vs. 8.9–17.4, P = 0.593) between EP and carboplatin-etoposide regimens (27), though an international survey by Lamarca et al. revealed significant intercenter heterogeneity in platinum-etoposide protocols, including variations in dosing, administration routes, and treatment cycles (28). Alternative gemcitabine-based regimens, typically used for biliary tract cancers, may also be considered. In our Case 4, where the tumor involved the gallbladder neck and common bile duct, the patient achieved 1-year PFS following pancreaticoduodenectomy and six cycles of GEMOX without additional adjuvant therapy. Notably, GB-NENs may exhibit heightened chemoresistance potential. RB1 mutations, frequent in GB-NECs but absent in carcinomas, correlate with chemoresistance (15). We recommend platinum-based regimens as first-line, with gemcitabine alternatives for non-responders or intolerant patients, while monitoring for resistance.

### 2.3 Other adjuvant therapies

A single-arm, open-label phase II trial conducted by Chen et al. demonstrated a 30% increase in objective response rate with GEMOX plus camrelizumab in biliary tract cancer,while prolonging both OS and PFS (29), prompting inquiry into immunotherapy combinations for GB-NENs. Peptide receptor radionuclide therapy (PRRT), well-established for G1/G2 gastroenteropancreatic NENs, now shows efficacy in somatostatin receptor imaging (SRI)-positive G3 NENs (30), suggesting potential applicability to GB-NECs. Genomic characterization of GB-NECs has revealed clinically actionable alterations. EMS has identified ALK mutations (TKI-sensitive) and other targets (MYC, ZFHX3, Wnt pathway) (15). While immune-targeted therapies currently serve as late-line options with limited efficacy, their integration with chemotherapy may enhance outcomes, particularly in consolidating surgical results and advancing therapeutic discovery.

NENs show different characteristics depending on their location. While pancreatic and appendiceal NETs are often well-differentiated (G1/G2), GB-NENs are typically diagnosed as NET G3/NEC (4, 31). Analysis of median OS data from the SEER database by Arvind et al. demonstrated significant survival disparities: patients with rectal (24.6 years), appendiceal (>30 years), lung (5.5 years) and pancreatic (3.6 years) NETs live significantly longer than those with GB-NENs (23.2 months) (19, 32). This difference may reflect the distinct age distribution patterns between NETs at different anatomical sites, with GB-NETs demonstrating significantly later onset (median age at diagnosis: 63 years) compared to appendiceal NETs (median age: 34 years) (4, 31) or NETs at other anatomical sites (33). Notably, although gallbladder cancer and GB-NENs share the same organ origin and may arise from common precursor lesions, their divergent biological evolution results in significantly different outcomes. GB-NEN patients exhibit substantially worse survival compared (3-year overall survival rate: 31.1% vs 63.8%, P<0.01) (34). These differences likely reflect that the differentiation status and pathological characteristics of NENs may reflect distinct tumor origins and molecular mechanisms of pathogenesis, which critically influence disease prognosis. Consequently, while treatment strategies for non-gallbladder NENs or gallbladder cancer may provide valuable references, therapeutic approaches for GB-NENs require specific modifications and innovations based on clinical response patterns. This adaptive approach will facilitate the development of novel, more effective treatment strategies tailored to the unique biology of GB-NENs. Moreover, emerging evidence supports circulating cytokines as dynamic biomarkers for assessing treatment response in NEN patients, facilitating adaptive therapeutic interventions. Concurrently, systemic inflammation markers (neutrophil-lymphocyte ratio, platelet-lymphocyte ratio, PD-1/PD-L1) show prognostic utility, advancing precision management strategies for GB-NENs (35).

## 3 Conclusion

Our case series examines GB-NENs patients receiving different treatments, sharing our center’s clinical experience while comparing management strategies with gallbladder cancer and NENs from other sites. Certainly, this study still has several important limitations: the extended timeframe introduces variability due to evolving diagnostic and surgical techniques, and as a single-center retrospective analysis with small sample size, our findings require validation through future multicenter prospective studies. These results should be viewed as preliminary experience rather than definitive evidence.

## Data Availability

The original contributions presented in the study are included in the article/**Supplementary Material**. Further inquiries can be directed to the corresponding author.
