# Supplementary figures and images for: Case Report: Diagnosing and treating gallbladder neuroendocrine neoplasms through comparative analysis: a case series and literature review
Source: Front Oncol. 2025 Jun 16;15:1606850. doi: 10.3389/fonc.2025.1606850 (PMC12206783; doi:10.3389/fonc.2025.1606850)

## Slide 1
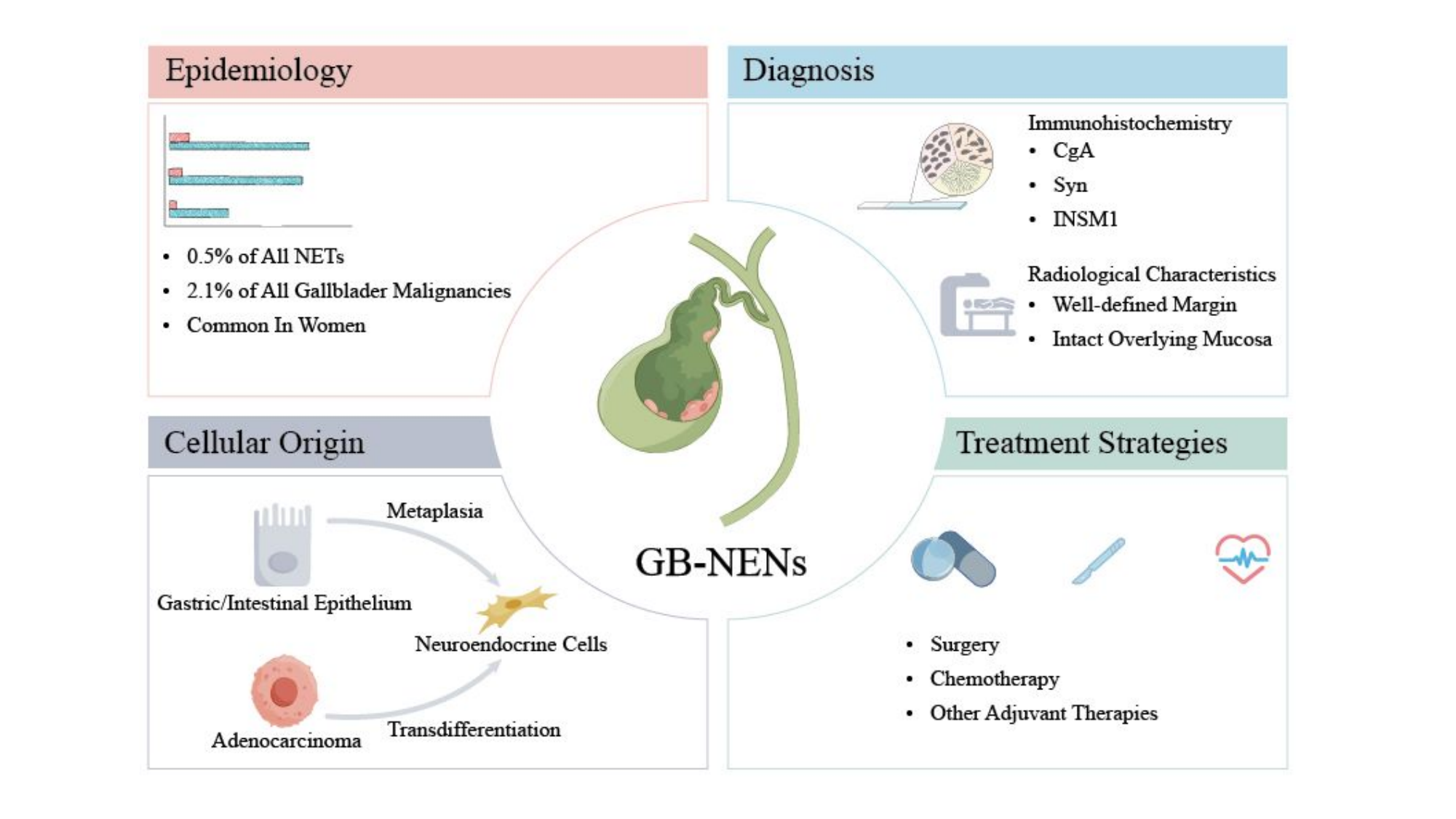

Supplement: Supplementary file 1 [file Supplementaryfile1.pptx]
